# Handedness for Unimanual Grasping in 564 Great Apes: The Effect on Grip Morphology and a Comparison with Hand Use for a Bimanual Coordinated Task

**DOI:** 10.3389/fpsyg.2015.01794

**Published:** 2015-11-23

**Authors:** Adrien Meguerditchian, Kimberley A. Phillips, Amandine Chapelain, Lindsay M. Mahovetz, Scott Milne, Tara Stoinski, Amanda Bania, Elizabeth Lonsdorf, Jennifer Schaeffer, Jamie Russell, William D. Hopkins

**Affiliations:** ^1^CNRS, Laboratory of Cognitive Psychology, UMR 7290, Brain and Language Research Institute, Aix-Marseille University, Marseille, France; ^2^Department of Psychology, Trinity University, San Antonio, TX, USA; ^3^CNRS, Laboratoire d’Éthologie Animale et Humaine EthoS, UMR 6552, Université de Rennes 1, Paimpont, France; ^4^Department of Psychology, Georgia State University, Atlanta, GA, USA; ^5^Department of Biology, Kennesaw State University, Kennesaw, GA, USA; ^6^The Dian Fossey Gorilla Fund International, Atlanta, GA, USA; ^7^Smithsonian’s National Zoo, Washington, DC, USA; ^8^Department of Psychology, Franklin and Marshall College, Lancaster, PA, USA; ^9^Division of Developmental and Cognitive Neuroscience, Yerkes National Primate Research Center, Atlanta, GA, USA; ^10^Neuroscience Institute, Georgia State University, Atlanta, GA, USA; ^11^Language Research Center, Georgia State University, Atlanta, GA, USA

**Keywords:** grasping, handedness, grip morphology, hemispheric specialization, primate

## Abstract

A number of factors have been proposed to influence within and between species variation in handedness in non-human primates. In the initial study, we assessed the influence of grip morphology on hand use for simple reaching in a sample of 564 great apes including 49 orangutans *Pongo pygmaeus*, 66 gorillas *Gorilla gorilla*, 354 chimpanzees *Pan troglodytes* and 95 bonobos *Pan paniscus*. Overall, we found a significant right hand bias for reaching. We also found a significant effect of the grip morphology of hand use. Grasping with the thumb and index finger was more prevalent in the right compared to left hand in all four species. There was no significant sex effect on the patterns of handedness. In a subsample of apes, we also compared consistency in hand use for simple reaching with previously published data on a task that measures handedness for bimanual actions. We found that the ratio of subjects with consistent right compared to left hand use was more prevalent in bonobos, chimpanzees and gorillas but not orangutans. However, for all species, the proportion of subjects with inconsistent hand preferences between the tasks was relatively high suggesting some measures may be more sensitive in assessing handedness than others.

## Introduction

All human populations have been shown to exhibit a predominance of right-handedness (Perelle and Ehrman, 1994; Abell et al., 1999; Annett, 2002; Raymond and Pontier, 2004), particularly for complex motor actions (Marchant et al., 1995; Fagard and Marks, 2000). Such lateralization is one of the main expressions of the hemispheric specialization of the human brain and presumably reflects a dominance of the left hemisphere for manual motor skills (Springer and Deutsch, 1993; Hammond, 2002). Whether population-level handedness can be dated back even further in Hominid evolution remains a topic of intense debate (MacNeilage et al., 1987; McGrew and Marchant, 1997; Hopkins, 2006; Cashmore et al., 2008; Uomini, 2009). Some authors argue that right-handedness is a hallmark of human evolution (Warren, 1980; Ettlinger, 1988; Crow, 2004), whereas there is a growing body of evidence of population-level behavioral and brain asymmetries in a host of vertebrates (MacNeilage et al., 2009; Rogers et al., 2013) including also some reports of population-level of right-handedness in non-human primates (Hopkins et al., 2007). Regarding the phylogenetic proximity between human and non-human primates, studying hand preferences within a comparative approach among primates might help evaluate different evolutionary models of handedness (Hopkins, 2013a).

In the non-human primate literature, the collective studies on handedness reveal divergent patterns of hand preference within and between the species but also show large variability concerning the method of data collection, sample size, environment of the subjects (e.g., captive vs. wild subjects), ecology of the species (arboreal vs. terrestrial species), and the manual tasks used for assessing hand preferences (see McGrew and Marchant, 1997; Papademetriou et al., 2005; Hopkins, 2007). Therefore, it remains unclear which factors primarily drive the expression of handedness in non-human primates. According to some authors, the inconsistent patterns of findings across the handedness literature in non-human primates reflect differences in the behavior measured and in the sample size of subjects (Hopkins, 1999, 2013a,b; Hopkins and Cantalupo, 2005). Indeed, there is a large body of evidence of the effect of the task complexity on the direction, magnitude and consistency of the individual hand preferences in humans (Perelle and Ehrman, 1994; Marchant et al., 1995; Fagard, 2001), great apes (Boesch, 1991; O’Malley and McGrew, 2006; Humle and Matsuzawa, 2009; Bogart et al., 2012), and monkeys (Fagot and Vauclair, 1988, 1991; Fagot et al., 1991; Spinozzi et al., 1998; Blois-Heulin et al., 2006; Lilak and Phillips, 2007; Meunier and Vauclair, 2007; Schweitzer et al., 2007). In most of these studies, the distinction between unimanual reaching actions and bimanual coordinated behaviors has been critical for the task complexity’s effects on individual hand preferences and for revealing population-level handedness. For instance, within the same sample populations, simple behavioral measures of hand preferences such as unimanual reaching have usually revealed an absence of or weak population-level hand bias compared to bimanual tasks in humans (Fagard and Marks, 2000), captive De Brazza’s monkeys (Schweitzer et al., 2007), wild Sichuan snub-nosed monkeys, *Rhinopithecus roxellana* (Zhao et al., 2010), wild mountain gorillas, *Gorilla g. beringei* (Byrne and Byrne, 1991), captive western lowland gorillas *Gorilla gorilla* (Meguerditchian et al., 2010), captive chimpanzees *Pan troglodytes* (Hopkins and Rabinowitz, 1997; Llorente et al., 2009), olive baboons *Papio anubis* (Vauclair et al., 2005), and capuchin monkeys *Cebus apella* (Spinozzi et al., 1998). Collectively, these results indicate that complex bimanual actions appear to be more sensitive for detecting hand preferences than unimanual tasks, such as unimanual reaching.

It has been also suggested that hand preferences for bimanual coordinated tasks might better reflect hemispheric lateralization of the brain than hand preference for unimanual tasks. For instance, hand preference for tasks that bimanual coordination but not unimanual reaching, have been shown to be more strongly related to morphologic inter-hemispheric asymmetries of the motor hand area of the precentral gyrus (Hopkins and Cantalupo, 2004; Phillips and Sherwood, 2005; Dadda et al., 2006). Finally, whereas in humans, some consistency of significant hand preferences have been shown between unimanual reaching and bimanual task (see Marchant et al., 1995; Fagard and Marks, 2000), most of the studies that have investigated both tasks in non-human primates have revealed an absence or weak correlations of measure of hand preference between unimanual reaching and bimanual coordinated task (e.g., in baboons: Vauclair et al., 2005; in squirrel monkeys: Meguerditchian et al., 2012; in capuchin monkeys: Fagot and Vauclair, 1988, 1991; Fagot et al., 1991; Spinozzi et al., 1998; Blois-Heulin et al., 2006; Lilak and Phillips, 2007; Meunier and Vauclair, 2007; Schweitzer et al., 2007; in chimpanzees: Hopkins, 2013a; Hopkins et al., 2013; but see Hopkins and Rabinowitz, 1997, for unimanual and bimanual tool use).

One potential explanation for the difference in handedness found between unimanual and bimanual tasks is that unimanual tasks might be more sensitive to environmental and situational factors such as position of the food than bimanual tasks (e.g., Meunier et al., 2011). For instance, it has been demonstrated in monkeys that the position of the target (i.e., food) in relation to the position of the subject influences the choice of the hand used for grasping an object (Lehman, 1993). In other word, an object at a left position from the subject would favor the use of the left hand. In contrast, it has been argued that the use of both hands to manipulate an object in a bimanual coordinated task minimizes such postural and situational biases for detecting hand preference (Hopkins, 1995).

Nevertheless, such a contrast of hand preference between unimanual and bimanual behaviors in primates has been challenged by some reports showing that unimanual reaching can reveal significant hand preference results when taking into account other mediating factors such as body posture and grip morphology. For instance, bipedal reaching elicited a greater right-hand preference than quadrupedal reaching in many primate species (Olson et al., 1990; Hopkins, 1993; Westergaard et al., 1998; Laurence et al., 2011) whereas “precision grasping” (i.e., using the thumb and the index finger for food reaching) elicited an increased bias toward right-handedness in captive great apes (Christel, 1994; Tonooka and Matsuzawa, 1995; Jones-Engel and Bard, 1996; Christel et al., 1998; Hopkins et al., 2002; Pouydebat et al., 2009, 2011). Other effects of grip morphology of reaching on the pattern of hand preference have been also reported in monkey species (Costello and Fragaszy, 1988; Spinozzi et al., 2004).

These latter findings in non-human primates, especially the great apes, indicate that unimanual reaching actions are pertinent to handedness and should be not excluded from investigation in primates within a larger comparative and evolutionary perspective on handedness. Moreover, the question of its relevancy for investigating landmark of hemispheric specialization of the brain has been revived by a recent functional and anatomical brain imaging study in chimpanzees. This study revealed that lateralized hand use for grasping was associated to contralateral brain asymmetries in the white matter of the motor hand area of the precentral gyrus (Hopkins et al., 2010), an anatomical correlate which overlaped with PET brain activation in this region. Thus the link between asymmetries in the motor hand area and handedness are in fact not exclusively related to tasks that require bimanual coordination as demonstrated in the previous neuroanatomical study in chimpanzees (Hopkins and Cantalupo, 2004). A previous report on hand preferences in 777 great apes highlighted the potential role of bimanual coordinated behaviors in the evolution of handedness (Hopkins et al., 2011). For unimanual reaching tasks, Papademetriou et al. (2005) conducted a meta-analysis on hand preferences in primates and concluded that there were inconsistent effects for population-level handedness in great apes. However, no systematic comparative data using an identical unimanual reaching task are available as yet across great apes species.

In the present study, we investigated hand preference for unimanual food reaching in a large captive sample of all great apes species: bonobos, orangutans, gorillas and chimpanzees. We subsequently analyzed the effect on the morphology of grasping on the pattern of hand preference in each species. The objective of the study was to comparatively investigate the potential role of within and between species variation in grasping morphology on asymmetries in reaching. We hypothesized that precision grasping, defined as the use of the thumb and index finger to pick up a small food item, would be more likely to elicit population-level handedness in great apes than other grasping morphology patterns. In addition, we evaluated potential sex effect on the patterns of handedness as it has been reported for unimanual reaching in some studies in non-great ape monkeys species (e.g., in squirrel monkeys: Meguerditchian et al., 2012; in red-capped mangabeys: Laurence et al., 2011; but not in the rest of the literature in great apes species Lehman, 1993; Hook-Costigan and Rogers, 1997; Papademetriou et al., 2005).

Additionally, we compared the patterns of hand preference for grasping with data previously reported for the bimanual coordinated tube task (Hopkins et al., 2011). The TUBE task consists of holding a PVC tube with one hand and extracting the food inside the tube with the fingers of the opposite (dominant) hand. This comparison allowed us to test the hypothesis that bimanual tasks elicit greater individual and population-level handedness than unimanual tasks in great apes. Finally, by combining the hand preference data for simple reaching and the TUBE task, we were able to assess consistency in hand use across the different species. Within an evolutionary framework, we believe this comparative approach using strong statistical power might help understand the potential factors that drive manual asymmetries in great apes as well as the phylogenetical precursors of the emergence and evolution of population-level handedness in the primate lineage.

## Materials and Methods

### Subjects

Hand preference data for unimanual reaching were collected on 564 captive great apes including 49 orangutans *Pongo pygmaeus* (26 females and 23 males), 66 gorillas *Gorilla gorilla* (31 females and 35 males), 354 chimpanzees *Pan troglodytes* (196 females and 158 males), and 95 bonobos *Pan paniscus* (53 females and 42 males). Morphology of the grip (see procedure section) was observed for a subset of 459 apes within each species including 47 bonobos, 329 chimpanzees, 54 gorillas, and 29 orangutans. Data were collected from several research facilities and zoos. The gorilla data were collected at the National, Milwaukee County, Lincoln Park, Columbus and Jacksonville zoos. The orangutan data were collected at the Ape Cognition and Conservation Initiative as well as the National, Cleveland, Columbus, Honolulu, and Toledo zoos. Bonobo handedness data were collected from Ape Cognition and Conservation Initiative, Lola Ya Bonobo sanctuary and the Jacksonville, Milwaukee County and Columbus zoos. Finally, chimpanzee data were collected at the Yerkes National Primate Research Center, University of Texas M D. Anderson Cancer Center and Honolulu zoo.

### Procedure

We recorded hand use for unimanual behaviors by observations of the social groups of apes. The observers chose a given focal subject and small food items such as raisins or peanuts were scattered throughout the subjects’ indoor or outdoor enclosure. The focal subject would move to different locations in the enclosure to grasp the food item. The experimenter would then record their hand use as left or right during each discrete reaching response. If multiple apes were feeding, the experimenter(s) picked up the focal subject as the one being in the most visible area of the enclosure and for which the fewest data points were available because some individuals (i.e., dominant apes) had greater access to the food and performed more grasping behaviors than others. In order to obtain a reasonable numbers of observations for each subject (about 50 responses whenever possible) and increase our overall sample size, a concerted effort was made to focus on subjects that had the fewest observations whenever possible. In order to minimize postural biases in the choice of the hand, to be considered a valid reaching response, the subject had to be in a symmetrical posture, either seated or quadrupedal, with both hands available and able to grasp the food in front of them. A single unimanual response was recorded for a reaching response and the subjects had to reposition themselves and move to another location between reaching responses in order to obtain discrete responses. As noted above, for a subset of 459 subjects including 47 bonobos, 329 chimpanzees, 54 gorillas and 29 orangutans, the experimenter also recorded whether or not unimanual reaching responses involved the thumb-index finger precision grasping (TI; using the thumb and index finger). Multiple observers were involved for coding the different groups of subjects and followed a consistent behavioral coding methodology as described above. In order to minimize potential ambiguity of coding across observers, we decided to not consider the variability of grip morphology other than the use of the thumb-index grip (TI). Any other grips or reaching responses that were ambiguous were then combined into a single category, namely non-thumb-index grip category (Non-TI). We used this constraint not only to increase statistical power but also to assure that grips, other than those using the thumb-index fingers, were as unambiguous as possible across observers. Such conservative constraints restricted the number of subjects for which we could reliably investigate the effect of grip type on patterns of hand use.

### Data Analysis

For determining individual hand preference, we used two different methods. First, the direction of hand preference for each subject was determined by calculating an individual *z*-score on the basis of their total left and right hand responses. Based on their *z*-score, the individual apes were classified as left-handed (*z* ≤ -1.96), right-handed (*z* ≥ 1.96), or ambiguously handed (–1.96 < *z* < 1.96). Second, the degree of hand preference was placed on a continuous scale of measurement by calculating an individual handedness index score (HI) using the formula (R – L)/(R + L), where R and L represent the total right and left hand responses, respectively. The HI values varied on a continuum from -1.0 (exclusive left hand use) to 1.0 (exclusive right hand use) with the sign indicating the direction of hand preferences (positive = right hand preference; negative = left hand preference). For each subject, we also calculated the percentage of TI and Non-TI grasping responses for the left and right hands by dividing the number of TI and Non-TI grips for each hand by the total number of responses and multiplying by 100. All data were analyzed using Statistical Package for the Social Sciences (SPSS) with alpha set to *p* < 0.05. Any necessary *post hoc* tests were conducted using Tukey’s Honestly Significant Difference test.

## Results

### Handedness for Unimanual Reaching

Descriptive data on handedness for each species are provided in Table 1. As noted above, overall hand preference data were available in 564 apes. Overall, a one-sample *t*-test on the HI scores revealed a significant rightward bias *t*(563) = 3.565, *p* < 0.001. Based on the classification data, there were 216 right-, 207 ambiguously-, and 141 left-handed apes, a distribution that differs significantly from a predicted random distribution *χ*^2^(2, *N* = 564) = 17.84, *p* < 0.001. The number of right-handed was significantly higher than the number of left-handed individuals *χ*^2^(1, *N* = 357) = 15.76, *p* < 0.001. The number of ambiguously-handed individuals was significantly higher than the number of left-handed, *χ*^2^(1, *N* = 348) = 12.52, *p* < 0.005 but did not differ from the number of right-handed apes, *χ*^2^(1, *N* = 423) = 0.19, *p* = 0.662.

**TABLE 1:** **Distribution of handedness and mean HI scores for unimanual reaching in each species**.

**Ape species**	**#L**	**#A**	**#R**	**HI**	**SE**	***t***	***p***	***d***
Pongo	12	17	20	0.070	0.056	1.308	0.197	0.378
Gorilla	10	28	28	0.147	0.044	3.297*	0.003	0.779
Chimpanzee	96	129	129	0.044	0.021	2.030*	0.043	0.216
Bonobo	23	33	39	0.053	0.041	1.420	0.159	0.292

*Indicates significant bias. Effect sizes were determined using Cohen’s d.

We next considered the effects of sex and species on the HI scores and handedness distribution. Because the HI scores were not normally distributed, we used non-parametric statistics for these analyses. A Kruskal–Wallis test failed to reveal significant species differences in ranked HI scores and a Mann–Whitney *U*-test revealed no significant differences between sexes. The results described above were largely confirmed when analyzing the hand preference classification data (see Table 1). No significant associations were found between hand preference classification and either sex or species. To assess whether population-level biases were evident within each species, we conducted one sample *t*-tests within each species. The mean AQ scores, *t*-scores, *p*-values and effect sizes are shown in Table 1. Gorillas and chimpanzees showed small but significant rightward biases while bonobos and orangutans did not show significant biases. Though all four species had a relatively large proportion of ambiguously-handed individuals, inspection of Table 1 shows that the proportion of right-to-left-handed individuals was much higher in gorillas and chimpanzees compared to all other species.

### Grasping Morphology and Hand Use

**FIGURE 1:**
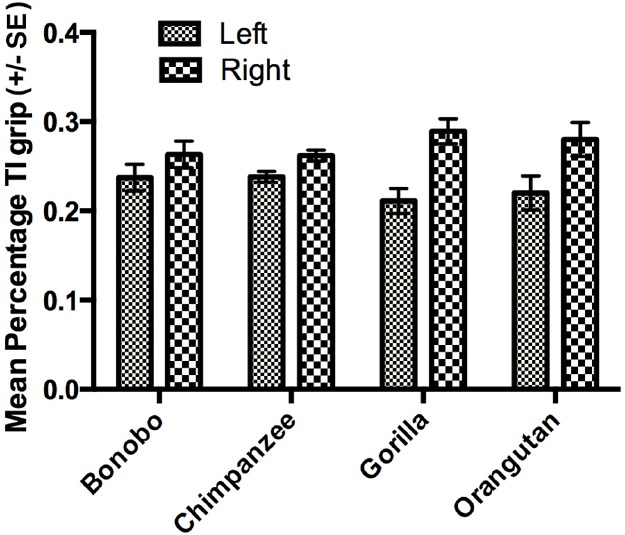
**Mean percentage TI grips (±SE) for the left and right hands in each species**.

**FIGURE 2:**
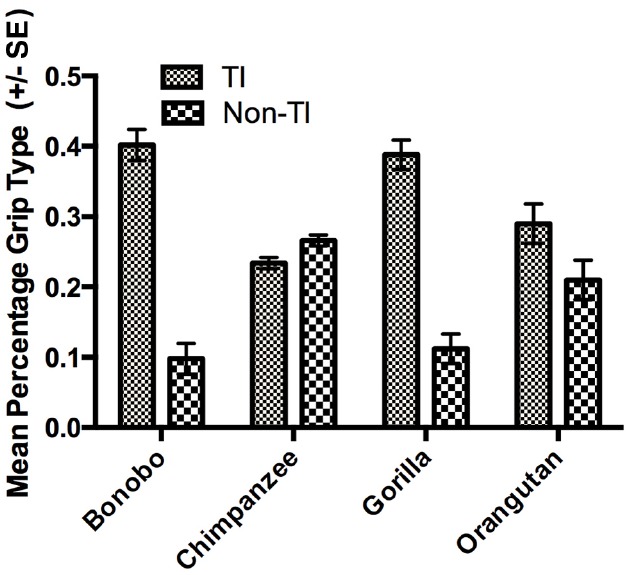
**Mean percentage of TI and Non-TI grips (±SE) for each species**.

The next set of analyses was restricted to those individuals for which we could reliably record their grasping morphology (TI or Non-TI). For this analysis, we used a mixed model analysis of variance (ANOVA) with the percentage TI and Non-TI responses for the left and right hand serving as the repeated measures while sex and species were the between group factors. Because ANOVA requires a normally distributed dependent variable, arcsine transformations were applied to percentage of TI and Non-TI grips for the left and right hand to correct for normality. Significant main effects were found for hand use *F*(1,448) = 8.230, *p* < 0.004 and species *F*(3,448) = 6.700, *p* < 0.001. We also found significant two-way interactions between grip type and sex *F*(1,448) = 4.900, *p* < 0.001 as well as grip type and species *F*(3,448) = 28.949, *p* < 0.001. The mean percentage of TI and Non-TI grips for the left and right hands for each species are shown in Figure 1. Overall, a higher percentage of TI grips were performed by the right compared to left hand. For species by grip type interaction, post-hoc analysis indicated that gorillas, bonobos, and orangutans produced a significantly higher percentage of TI compared to Non-TI grips while no difference in the percentage of the different grip types was found in the chimpanzees (see Figure 2). Finally, for the interaction between grip type and sex, males produced a higher percentage of TI grips (Mean = 35.0, SE = 1.50) than female TI (Mean = 30.7, SE = 0.1.40), male Non-TI (Mean = 15.0, SE = 1.50) and female Non-TI (Mean = 19.3, SE = 1.40) responses.

Lastly, we compared the HI scores for the reaching responses that were made with either the TI and Non-TI grip types. For this analysis, HI scores were derived for all subjects that made a minimum of six TI and Non-TI grip responses. The HI scores were then compared between sex and species using a mixed model ANOVA. No significant main effects or interactions were found. However, one sample *t*-tests on the HI scores did reveal that the apes showed a significant rightward bias for the TI *t*(293) = 2.694, *p* < 0.005 but not the Non-TI grip types *t*(293) = 1.209, *p* = 0.228.

### Comparison of Hand Preferences with Bimanual Coordinated Task Measures

**FIGURE 3:**
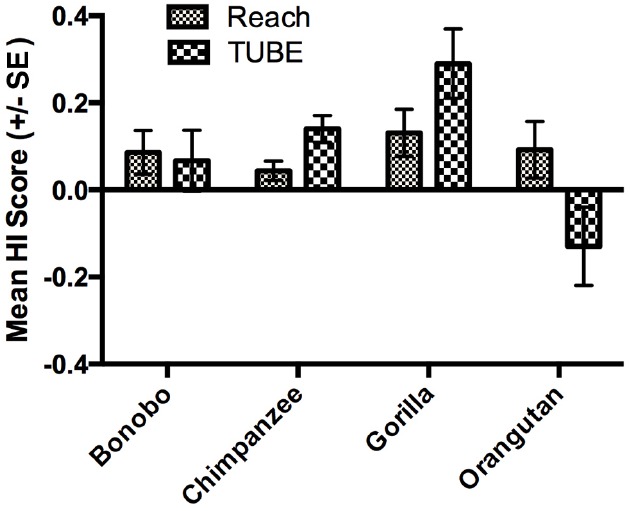
**Mean HI scores (±SE) for the TUBE and Reaching tasks in each species.** The sign of Mean HI scores indicates the direction of the manual bias (negative value: left-hand bias, positive value: right-hand bias).

As mentioned in the introduction, measures of hand preferences for bimanual coordinated activities have been collected using an experimental tube task (TUBE) in a number of the great apes tested in the present study (Hopkins et al., 2011). Specifically, data on the TUBE and reaching task were available in 514 apes including 70 bonobos, 347 chimpanzees, 57 gorillas and 40 orangutans, respectively. Among the TUBE task sample, 251 subjects were classified by *z*-score as right-handed, 169 as left-handed and 94 as ambiguously-handed. The availability of the TUBE and unimanual reaching data allowed us to evaluate consistency and variability in hand use in relation to these tasks. In the initial analysis, we compared the HI scores for the reaching and TUBE tasks using a mixed model ANOVA with task (TUBE, reaching) serving as the repeated measure while sex and species were the between group factors. A main effect for species *F*(3,505) = 3.131, *p* < 0.03 was found as well as a two-way interaction between species and task *F*(3,505) = 3.741, *p* < 0.02. The mean HI scores for each task and species are shown in Figure 3. *Post hoc* analysis indicated that chimpanzees had significantly higher HI scores for the TUBE compared to reaching task. In contrast, orangutans had significantly lower HI scores on the TUBE task compared to unimanual reaching. No between task difference in HI scores were found for bonobos and gorillas. It is also of note that gorillas showed the highest combined HI scores compared to all other apes.

As a means of further testing consistency in hand use, we performed a second analysis. For this test, we assigned values of 1, 2, or 3 to subjects classified as left-, ambiguously- and right-handed for both the reaching and TUBE task. We then combined the weighted values for the two tasks resulting in a range of scores from 2 (left-handed for both measures, LL) to 6 (right-handed for both measures, RR; see Table 2). The distribution of the combined handedness scores was then compared between sexes and species using a chi-square test of independence. No significant association was found between consistency in hand use and sex; however, there was a significant association between species and consistency in hand use *χ*^2^(12, *N* = 514) = 24.863, *p* < 0.02 (see Table 2). As can be seen, the proportion of RR to LL subjects is twice as high in bonobos (14 vs. 7), gorillas (11 vs. 4) and chimpanzees (78 vs. 40) but not orangutans (4 vs. 3). Among the lateralized (left- vs. right-handed) subjects for the tube task that performed also unimanual task, we compared their respective HI scores measured for unimanual reaching. The group of right-handed subjects for the tube task has a greater mean HI score (more rightward) for unimanual reaching than the group of left-handed subjects, *t*-test, *t*(367) = 3.835, *p* < 0.001. Conversely, when comparing the respective HI scores measured for the tube task between the right-handed vs. left-handed groups of subjects for the unimanual task, the group of right-handed for unimanual reaching has a greater Mean HI score (more rightward) for the tube task than the left-handed subjects, *t*(220) = 3.301, *p* < 0.001. These results indicate some degree of consistency at the individual-level across the two tasks.

**TABLE 2:** **Distribution of consistency in hand preference between species**.

	**Pongo**	**Gorilla**	**Chimpanzee**	**Bonobo**
***Handedness score***				
LL	3	4	40	7
AL	9	3	56	12
AA or RL	16	12	94	20
AR	8	27	79	17
RR	4	11	78	14

LL, prefer left hand for both task; RR, prefer right hand for both tasks; RL, prefer opposite hands for the two tasks; AR, prefer right hand for one task, no preference for the other; AL, prefer left hand for one task and no preference for the other; AA, no hand preference for either task.

## Discussion

Three main results emerged from this study. First, overall, great apes showed a significant right-hand bias for unimanual reaching. Consistent with previous meta-analyses of handedness for simple reaching, we confirmed the absence of significant sex effects on the patterns of handedness. Though a majority of apes (51%) failed to show a significant hand preference in unimanual reaching, for those individuals that showed a significant hand preference, the number of right-handed individuals was significantly greater than the number of left-handed apes. Second, the degree of right-handedness for unimanual reaching was greater when the apes adopted a thumb-index grasping response compared to other grip types, the difference was small. Third, we found that males were more likely to produce a thumb-index grasping response than females and that gorillas and bonobos produced significantly more thumb-index compared to other grip responses than chimpanzees and orangutans.

Fourth, unimanual reaching elicited much more ambiguously-handed subjects (51%) than the TUBE task (20%) and significantly lower population-level handedness in chimpanzees and orangutans compared to gorillas and bonobos. However, when consistent direction of population-level hand preferences were found between the two tasks, bonobos, gorillas and chimpanzees showed a significant prevalence for right compared to left handedness. In contrast, orangutans showed no consistent pattern of population-level handedness between the TUBE (toward left-handedness) and reaching task (toward right-handedness). The evidence presented here showing that unimanual reaching induces weak lateralization at the individual and population-level is consistent with numerous previous studies in monkeys and apes (Lehman, 1993; Hook-Costigan and Rogers, 1997; Papademetriou et al., 2005). This suggests that unimanual reaching does not elicit a particularly strong or consistent measure of handedness in non-human primates likely due to the fact it is subject to influence by a variety of situational factors such as the position of the object relative to the subject (Tonooka and Matsuzawa, 1995; Hopkins, 2013a). Based on the findings reported here and elsewhere, it seems that the tasks requiring coordinated bimanual actions elicit stronger individual and population-level hand preferences in non-human primates, including great apes. These collective findings are consistent to what has been reported in developing human children although as soon as grasping emerges there are clear signs of hand preference in favor of the use of the right-hand in a majority of infants (e.g., Fagard and Marks, 2000; Ferre et al., 2010).

We further showed in this study that grasping morphology when reaching for a small food item differed between species and hands and that only TI grips elicited a predominance of right-handedness. Moreover, similar effect of fine precision grip on the degree of right-handedness has been found in young human infants for bimanual tasks (Potier et al., 2013), indicating that grip morphology including to use of single digit in precision movements is an important factor in the expression of individual hand preferences (Maille et al., 2013; Nelson and Boeving, 2015). Consistent with some previous reports (Christel, 1994; Christel et al., 1998; Pouydebat et al., 2011), gorillas and bonobos showed the highest percentages in thumb-index grasping responses compared to chimpanzees and orangutans. Further, all four species preferred thumb-index grasping with the right compared to left hand. Though increased thumb-index responses have been previously reported in chimpanzees (Tonooka and Matsuzawa, 1995; Jones-Engel and Bard, 1996; Hopkins and Russell, 2004; Hopkins et al., 2005), this effect appears to also be evident in other ape species.

The overall between species differences in grasping morphology are somewhat difficult to interpret based on what are known about the morphology of the hands in great apes, such as the distance between the thumb and tip of the index finger, or opposability index (Napier, 1980). On the one hand, gorillas have the highest opposability index of the great apes and thus it makes sense that they would prefer to use these two digits for grasping; however, chimpanzees and bonobos have similar hand morphology and opposability indices yet bonobos were much more prone to adopt a thumb-index grasping responses compared to chimpanzees. Why this was the case is not clear but warrants further investigation but given that our subjects all grasped the same types of food items and picked them up off similar surfaces, we do not think the effects are attributable to these types of variables.

When considering consistency in hand use, although some degree of consistency of hand preference was found between the reaching and bimanual TUBE task, a substantial number of the apes were inconsistent in their hand use. For those apes that showed consistent hand preferences between the two tasks, the bonobos, gorillas, and chimpanzees showed a higher proportion of right-compared to left-handed individuals. The opposite finding reported in orangutans specifically—the only arboreal species of the present study—is consistent with previous handedness studies conducted in orangutans (Rogers and Kaplan, 1996) and in other arboreal primates such as squirrel monkeys (Meguerditchian et al., 2012), red-capped mangabeys (Laurence et al., 2011), snub-nosed monkeys (Zhao et al., 2012) Brazza’s monkeys (Schweitzer et al., 2007), and spider monkeys (Nelson et al., 2105). Like in orangutans, these arboreal species show manual preferences that are more leftward for the TUBE task compared to simple reaching or similar unimanual tasks. On the other hand, when considering both tasks together, gorillas showed the most robust and consistent level of right-handedness (see Figure 3). There are at least two important points of interpretation of these results. First, though it has been previously claimed that gorillas are more right-handed than other apes, McGrew and Marchant (1993) reviewed the evidence of handedness on gorillas and concluded that there was no evidence in support of this claim. One limitation of the McGrew and Marchant (1993) review was that they considered hand preferences across a variety of different tasks. In this study, consistency in hand use was assessed on the same tasks for all species and, under these conditions, gorillas appear to be more right-handed than chimpanzees, bonobos, and orangutans (see also Forrester et al., 2011, 2012, 2013). Why gorillas are more right-handed than other apes is unclear but, it is of note, that gorillas frequently engage in bimanual feeding (Byrne and Byrne, 1993) and are more habitually terrestrial than other apes, particularly orangutans. Posture and role differentiation of the hands related to ecological and biomechanical factors (e.g., living in aboreal vs. terrestrial conditions) have been hypothesized as important factors in the expression and evolution of handedness in the primate lineage and the findings reported here are consistent with these views (Wundrum, 1986; MacNeilage et al., 1987; Van Schaik et al., 1999).

Second, the large number of inconsistently handed individuals in all four apes might be interpreted as evidence in support of the claim that non-human primates exhibit task specific handedness whereas human handedness is more consistent across different tasks, or true handedness (McGrew and Marchant, 1997). This very well might be the case but we are somewhat cautious of this explanation for two reasons. First, we only used two measures and this may not be enough to capture consistency. Additionally, recall that unimanual reaching did not elicit consistent hand preferences in a majority of individual within each species. Thus, as noted above, it not a particularly sensitive measure of hand preference. It is quite possible and, indeed likely, that consistency in hand use would be much higher had all the tasks elicited stronger hand preferences at the individual level (Hopkins, 2013a; Hopkins et al., 2013) and required similar motor demands.

In summary, this study provides additional support not only to the view that unimanual reaching is a poor marker of lateralization compared to coordinated bimanual actions in great apes but also that the morphology of the grip and the ecology of the species are important factors in the expression of handedness. These critical distinctions between (1) unimanual vs. bimanual coordinated task, (2) fine grip vs. other grip, and (3) arboreal vs. terrestrial species should be taken into account in further studies on hand preferences. The consideration of these factors within a comparative approach among primates including humans might further help developing model on the evolution of handedness among the primates lineage and better delimitate the continuities/discontinuities between non-human and human primates concerning handedness and the hemispheric specialization of the brain.

### Conflict of Interest Statement

The authors declare that the research was conducted in the absence of any commercial or financial relationships that could be construed as a potential conflict of interest.

## References

[B1] Abell F. Krams M. Ashburner J. Passingham R. Friston K. Frackowiak R. (1999). The neuroanatomy of autism: a voxel-based whole brain analysis of structural scans. Neuroreport 10, 1647–1651. 10.1097/00001756-199906030-00005 10501551

[B2] Annett M. (2002). Handedness and Brain Asymmetry: The Right Shift Theory. Hove: Psychology Press.

[B3] Blois-Heulin C. Guitton J. S. Nedellec-Bienvenue D. Ropars L. Vallet E. (2006). Hand preference in unimanual and bimanual tasks and postural effect on manual laterality in captive red-capped mangabeys (*Cercocebus torquatus torquatus*). Am. J. Primatol. 68, 429–444. 10.1002/ajp.20239 16541442

[B4] Boesch C. (1991). Handedness in wild chimpanzees. Int. J. Primatol. 6, 541–558. 10.1007/BF02547669

[B5] Bogart S. L. Pruetz J. D. Ormiston L. K. Russell J. L. Meguerditchian A. Hopkins W. D. (2012). Termite fishing laterality in the fongoli savanna chimpanzees (*Pan troglodytes verus*): further evidence of a left hand preference. Am. J. Phys. Anthropol. 149, 591–598. 10.1002/ajpa.22175 23129227

[B6] Byrne R. W. Byrne J. M. (1991). Hand preferences in the skilled gathering tasks of mountain gorillas (*Gorilla gorilla beringei*). Cortex 27, 521–536. 10.1016/S0010-9452(13)80003-2 1782788

[B7] Byrne R. W. Byrne J. M. (1993). Complex leaf-gathering skills of Mountain Gorillas (*Gorilla g. beringei*): variability and standardization. Am. J. Primatol. 31, 241–261. 10.1002/ajp.1350310402 31936992

[B8] Cashmore L. Uomini N. Chapelain A. (2008). The evolution of handedness in humans and great apes: a review and current issues. J. Anthropol. Sci. 86, 7–35. 19934467

[B9] Christel M. I. (1994). “Catarrhine primates grasping small objects: techniques and hand preferences,” in Current Primatology, Vol. 3, *Behavioral Neuroscience, Physiology and Reproduction*, eds Anderson J. R. Roeder J. J. Thierry B. Herrenschmidt N. (Strasbourg: Universite Louis Pasteur), 37–49.

[B10] Christel M. I. Kitzel S. Niemitz C. (1998). How precisely do bonobos (*Pan paniscus*) grasp small objects? Int. J. Primatol. 19, 165–194. 10.1023/A:1020319313219

[B11] Costello, M. and Fragaszy D. M. (1988). Prehension in Cebus and Samiri: I. Grip type and hand preference Am. J. Primatol. 15, 234–245. 10.1002/ajp.1350150306 31968893

[B12] Crow T. (2004). Directional asymmetry is the key to the origin of modern *Homo sapiens* (the Broca-Annett axiom): a reply to Rogers’ review of *The Speciation of Modern Homo Sapiens*. Laterality 9, 233–242. 10.1080/13576500342000374

[B13] Dadda M. Cantalupo C. Hopkins W. D. (2006). Further evidence of an association between handedness and neuroanatomical asymmetries in the primary cortex of chimpanzees (*Pan troglodytes*). Neuropsychologia 44, 2482–2486. 10.1016/j.neuropsychologia.2006.03.037 16730360 PMC2025584

[B14] Ettlinger G. F. (1988). Hand preference, ability and hemispheric specialization. How far are these factors related in the monkey? Cortex 24, 389–398. 10.1016/S0010-9452(88)80002-9 3191723

[B15] Fagard J. (2001). “Le développement de la latéralité manuelle,” in Le développement des habiletés de l’enfant. Coordination bimanuelle et latéralité, ed. Fagard J. (Paris: CNRS Editions), 221–234.

[B16] Fagard J. Marks A. (2000). Unimanual and bimanual tasks and the assessment of handedness in toddlers. Dev. Sci. 3, 137–147. 10.1111/1467-7687.00107

[B17] Fagot J. Drea C. Wallen K. (1991). Asymmetrical hand use in rhesus monkeys (*Macaca mulatta*) in tactually and visually regulated tasks. J. Comp. Psychol. 105, 260–268. 10.1037/0735-7036.105.3.260 1935005

[B18] Fagot J. Vauclair J. (1988). Handedness and bimanual coordination in the lowland gorilla. Brain Behav. Evol. 32, 89–95. 10.1159/000116536 3179697

[B19] Fagot J. Vauclair J. (1991). Manual laterality in nonhuman primates: a distinction between handedness and manual specialization. Psychol. Bull. 109, 76–89. 10.1037/0033-2909.109.1.76 2006230

[B20] Ferre C. L. Babik I. Michel G. F. (2010). Development of infant prehension handedness: a longitudinal analysis during the 6- to 14-month age period. Infant Behav. Dev. 33, 492–502. 10.1016/j.infbeh.2010.06.002 20619463

[B21] Forrester G. S. Leavens D. A. Quaresmini C. Vallortigara G. (2011). Target animacy influences gorilla handedness. Anim. Cogn. 14, 903–907. 10.1007/s10071-011-0413-6 21562817

[B22] Forrester G. S. Quaresmini C. Leavens D. A. Mareschal D. Thomas M. S. C. (2013). Human handedness: an inherited evolutionary trait. Behav. Brain Res. 237, 200–206. 10.1016/j.bbr.2012.09.037 23022751

[B23] Forrester G. S. Quaresmini C. Leavens D. A. Spiezio C. Vallortigara G. (2012). Target animacy influences chimpanzee handedness. Anim. Cogn. 15, 1121–1127. 10.1007/s10071-012-0536-4 22829099

[B24] Hammond G. (2002). Correlates of human handedness in primary motor cortex: a review and hypothesis. Neurosci. Biobehav. Rev. 26, 285–292. 10.1016/S0149-7634(02)00003-9 12034131

[B25] Hook-Costigan M. A. Rogers L. J. (1997). Hand preferences in New World primates. Int. J. Comp. Psychol. 9, 173–207.

[B26] Hopkins W. D. (1993). Posture and reaching in chimpanzees (*Pan troglodytes*) and orangutans (*Pongo pygmaeus*). J. Comp. Psychol. 17, 162–168. 10.1037/0735-7036.107.2.162 8370269

[B27] Hopkins W. D. (1995). Hand preferences for a coordinated bimanual task in 110 chimpanzees: Cross-sectional analysis. J. Comp. Psychol. 109, 291–297. 7554825 10.1037/0735-7036.109.3.291

[B28] Hopkins W. D. (1999). On the other hand: statistical issues in the assessment and interpretation of hand preference data in non-human primates. Int. J. Primatol. 20, 851–866. 10.1023/A:1020822401195

[B29] Hopkins W. D. (2006). Comparative and familial analysis of handedness in great apes. Psychol. Bull. 132, 538–559. 10.1037/0033-2909.132.4.538 16822166 PMC2063575

[B30] Hopkins W. D. (2007). Evolution of Hemispheric Specialization in Primates. Oxford: Elsevier.

[B31] Hopkins W. D. (2013a). Comparing human and nonhuman primate handedness: challenges and a modest proposal for concensus. Dev. Psychobiol. 55, 621–636. 10.1002/dev.21139 23913784 PMC4041077

[B32] Hopkins W. D. (2013b). Independence of data points in the measurement of handedness: statistical problem or urban myth? Am. J. Phys. Anthropol. 151, 151–157. 10.1002/ajpa.22248 23460350 PMC3631286

[B33] Hopkins W. D. Cantalupo C. (2004). Handedness in chimpanzees is associated with asymmetries in the primary motor but not with homologous language areas. Behav. Neurosci. 118, 1176–1183. 10.1037/0735-7044.118.6.1176 15598127 PMC2043153

[B34] Hopkins W. D. Cantalupo C. (2005). Individual and setting differences in the hand preferences of chimpanzees (*Pan troglodytes*): a critical analysis and some alternative explanations. Laterality 10, 65–80. 10.1080/13576500342000301 15841824 PMC2147717

[B35] Hopkins W. D. Cantalupo C. Wesley M. J. Hostetter A. B. Pilcher D. (2002). Grip morphology and hand use in chimpanzees (*Pan troglodytes*): evidence of a left hemisphere specialization in motor skill. J. Exp. Psychol. Gen. 131, 412–423. 10.1037/0096-3445.131.3.412 12214755 PMC2080773

[B36] Hopkins W. D. Gardner M. Mingle M. Reamer L. Schapiro S. J. (2013). Within- and between-task consistency in hand use as a means of characterizing hand preferences in captive chimpanzees (*Pan troglodytes*). J. Comp. Psychol. 127, 380–391. 10.1037/a0031071 23356440 PMC3842357

[B37] Hopkins W. D. Phillips K. A. Bania A. Calcutt S. E. Gardner M. Russell J. L. (2011). Hand preferences for coordinated bimanual actions in 777 great apes: implications for the evolution of handedness in hominins. J. Hum. Evol. 60, 605–611. 10.1016/j.jhevol.2010.12.008 21334723 PMC3068228

[B38] Hopkins W. D. Rabinowitz D. M. (1997). Manual specialization and tool-use in captive chimpanzees (*Pan troglodytes*): the effect of unimanual and bimanual strategies on hand preference. Laterality 2, 267–277. 15513068 10.1080/713754273 PMC2039876

[B39] Hopkins W. D. Russell J. L. (2004). Further evidence of a right hand advantage in motor skill by chimpanzees (*Pan troglodytes*). Neuropsychologia 42, 990–996. 10.1016/j.neuropsychologia.2003.11.017 14998713

[B40] Hopkins W. D. Russell J. L. Hook M. Braccini S. Schapiro S. J. (2005). Simple reaching is not so simple: association between hand use and grip preferences in captive chimpanzees. Int. J. Primatol. 26, 259–277. 10.1007/s10764-005-2924-y 18163152 PMC2156199

[B41] Hopkins W. D. Russell J. L. Lambeth S. Schapiro S. J. (2007). “Handedness and neuroanatomical asymmetries in captive chimpanzees: a summary of 15 years of research,” in Evolution of Hemispheric Specialization in Primates, ed. Hopkins W. D. (London: Academic Press), 112–135.

[B42] Hopkins W. D. Taglialatela J. P. Russell J. L. Nir T. Schaeffer J. A. (2010). Cortical representation of lateralized grasping in chimpanzees (*Pan troglodytes*): a combined MRI and PET study. PLoS ONE 5:e13383. 10.1371/journal.pone.0013383 20967216 PMC2954174

[B43] Humle T. Matsuzawa T. (2009). Laterality in hand use across four tool use behaviors among the wild chimpanzees of Bossou, Guinea, West Africa. Am. J. Primatol. 71, 40–48. 10.1002/ajp.20616 18942096

[B44] Jones-Engel L. E. Bard K. A. (1996). Precision grips in young chimpanzees. Am. J. Primatol. 39, 1–15. 10.1002/(SICI)1098-2345(1996)39:1<1::AID-AJP1>3.0.CO;2-2 31918490

[B45] Laurence A. Wallez C. Blois-Heulin C. (2011). Task complexity, posture, age, sex: which is the main factor influencing manual laterality in captive *Cercocebus torquatus torquatus*? Laterality 16, 586–606. 10.1080/1357650X.2010.501338 21298589

[B46] Lehman R. A. W. (1993). “Manual preference in prosimians, monkeys, and apes,” in Primate Laterality: Current Behavioral Evidence of Primate Asymmetries, eds Ward J. P. Hopkins W. D. (New York: Springer-Verlag), 107–124.

[B47] Lilak A. L. Phillips K. A. (2007). Consistency in hand preference across low-level and high-level tasks in capuchin monkeys (*Cebus apella*). Am. J. Primatol. 69, 1–12. 10.1002/ajp.20485 17894405

[B48] Llorente M. Mosquera M. Fabre M. (2009). Manual laterality for simple reaching and bimanual coordinated task in naturalistic housed *Pan troglodytes* Int. J. Primatol. 30, 183–197. 10.1007/s10764-009-9338-1

[B49] MacNeilage P. F. Rogers L. J. Vallortigara G. (2009). Evolutionary origins of your right and left brain. Sci. Am. 301, 60–67. 10.1038/scientificamerican0709-60 19555025

[B50] MacNeilage P. F. Studdert-Kennedy M. G. Lindblom B. (1987). Primate handedness reconsidered. Behav. Brain Sci. 10, 247–303. 10.1017/S0140525X00047695

[B51] Maille A. Belboc’h C. Rossard A. Bec P. Blois-Heulin C. (2013). Which are the features of the TUBE task that make it so efficient in detecting manual asymmetries? An investigation in two cercopithecine species (*Cercopithecus neglectus* and *Cercocebus torquatus*). J. Comp. Psychol. 127, 436–444. 10.1037/a0032227 23772799

[B52] Marchant L. F. McGrew W. C. Eibl-Eibesfeldt I. (1995). In human handedness universal? Ethological analyses from three traditional cultures. Ethology 101, 239–258. 10.1111/j.1439-0310.1995.tb00362.x

[B53] McGrew W. C. Marchant L. F. (1993). Are gorillas right-handed or not? Hum. Evol. 8, 17–23. 10.1007/BF02436462

[B54] McGrew W. C. Marchant L. F. (1997). On the other hand: current issues in and meta-analysis of the behavioral laterality of hand function in non-human primates. Yearb. Phys. Anthropol. 40, 201–232.

[B55] Meguerditchian A. Calcutt S. E. Lonsdorf E. V. Ross S. R. Hopkins W. D. (2010). Captive gorillas are right-handed for bimanual feeding. Am. J. Phys. Anthropol. 141, 638–645. 10.1002/ajpa.21244 20033918 PMC2909605

[B56] Meguerditchian A. Donnot J. Molesti S. Francioly R. Vauclair J. (2012). Sex difference in squirrel monkeys’ handedness for unimanual and bimanual tasks. Anim. Behav. 83, 635–643. 10.1016/j.anbehav.2011.12.005

[B57] Meunier H. Blois-Heulin C. Vauclair J. (2011). A new tool for measuring hand preference in non-human primates: adaptation of Bishop’s Quantifying Hand Preference task for olive baboons. Behav. Brain Res. 218, 1–7. 10.1016/j.bbr.2010.11.011 21074568

[B58] Meunier H. Vauclair J. (2007). Hand preferences on unimanual and bimanual tasks in white-face capuchins. Am. J. Primatol. 69, 1064–1069. 10.1002/ajp.20437 17407149

[B59] Napier J. (1980). Hands. Princeton, NJ: Princeton University Press.

[B60] Nelson E. L. Boeving E. R. (2015). Precise digit use increases the expression of handedness in Colombian spider monkeys (*Ateles fusciceps rufiventris*). Am. J. Primatol. 77, 1253–1262. 10.1002/ajp.22478 26339782

[B61] Nelson E. L. Figueroa A. Albright S. N. Gonzalez M. F. (2105). Evaluating handedness measures in spider monkeys. Anim. Cogn. 18, 345–353. 10.1007/s10071-014-0805-5 25204683

[B62] Olson D. A. Ellis J. E. Nadler R. D. (1990). Hand preferences in captive gorillas, orangutans, and gibbons. Am. J. Primatol. 20, 83–94. 10.1002/ajp.1350200203 31963993

[B63] O’Malley R. C. McGrew W. C. (2006). Hand preferences in captive orangutans (*Pongo pygmaeus*). Primates 47, 279–283. 10.1007/s10329-006-0180-1 16604276

[B64] Papademetriou E. Sheu C. F. Michel G. F. (2005). A meta-analysis of primate hand preferences for reaching and other hand-use measures. J. Comp. Psychol. 119, 33–48. 10.1037/0735-7036.119.1.33 15740428

[B65] Perelle I. B. Ehrman L. (1994). An international study of human handedness: the data. Behav. Genet. 24, 217–227. 10.1007/BF01067189 7945152

[B66] Phillips K. A. Sherwood C. C. (2005). Primary motor cortex asymmetry is correlated with handedness in capuchin monkeys (*Cebus apella*). Behav. Neurosci. 119, 1701–1704. 10.1037/0735-7044.119.6.1701 16420175

[B67] Potier C. Meguerditchian A. Fagard J. (2013). Handedness for bimanual coordinated actions in infants as a function of grip morphology. Laterality 18, 576–593. 10.1080/1357650x.2012.732077 23231501

[B68] Pouydebat E. Reghem E. Borel A. Gorce P. (2011). Diversity of grip in adults and young humans and chimpanzees (*Pan troglodytes*). Behav. Brain Res. 218, 21–28. 10.1016/j.bbr.2010.11.021 21074572

[B69] Pouydebat W. Gorce P. Coppens Y. Bels V. (2009). Biomechanical study of grasping according to the volume of the object: human versus non-human primates. J. Biomech. 42, 266–272. 10.1016/j.jbiomech.2008.10.026 19100551

[B70] Raymond M. Pontier D. (2004). Is there geographical variation in human handedness? Laterality 9, 35–51. 10.1080/13576500244000274 15382729

[B71] Rogers L. J. Kaplan G. (1996). Hand preferences and other lateral biases in rehabilitated orangutans, *Pongo pygmaeus pygmaeus*. Anim. Behav. 51, 13–25. 10.1006/anbe.1996.0002

[B72] Rogers L. J. Vallortigara G. Andrew R. J. (2013). Divided Brains: The Biology and Behaviour of Brain Asymmetries. New York: Cambridge University Press.

[B73] Schweitzer C. Bec P. Blois-Heulin C. (2007). Does the complexity of the task influence laterality in De Brazza’s monkeys (*Cercopithecus neglectus*)? Ethology 113, 993–994. 10.1111/j.1439-0310.2007.01405.x

[B74] Spinozzi G. Castornina M. G. Truppa V. (1998). Hand preferences for unimanual and coordinated-bimanual tasks in tufted capuchin monkeys (*Cebus apella*). J. Comp. Psychol. 112, 183–191. 10.1037/0735-7036.112.2.183

[B75] Spinozzi G. Truppa V. Lagana T. (2004). Grasping behavior in tufted capuchin monkeys (*Cebus apella*): grip types and manual laterality for picking up a small food item. Am. J. Phys. Anthropol. 125, 30–41. 10.1002/ajpa.10362 15293329

[B76] Springer S. P. Deutsch G. (1993). Left Brain, Right Brain. New York: Freeman.

[B77] Tonooka R. Matsuzawa T. (1995). Hand preferences in captive chimpanzees (*Pan troglodytes*) in simple reaching for food. Int. J. Primatol. 16, 17–34. 10.1007/BF02700151

[B78] Uomini N. T. (2009). The prehistory of handedness: archeological data and comparative ethology. J. Hum. Evol. 57, 411–419. 10.1016/j.jhevol.2009.02.012 19758680

[B79] Van Schaik C. P. Deaner R. O. Merrill M. Y. (1999). The conditions for tool use in primates: implications for the evolution of material culture. J. Hum. Evol. 36, 719–741. 10.1006/jhev.1999.0304 10330335

[B80] Vauclair J. Meguerditchian A. Hopkins W. D. (2005). Hand preferences for unimanual and coordinated bimanual tasks in baboons (*Papio anubis*). Cogn. Brain Res. 25, 210–216. 10.1016/j.cogbrainres.2005.05.012 15993042 PMC2025585

[B81] Warren J. M. (1980). Handedness and laterality in humans and other animals. Physiol. Psychol. 8, 351–359. 10.3758/BF03337470

[B82] Westergaard G. C. Kuhn H. E. Suomi S. J. (1998). Bipedal posture and hand preference in humans and other primates. J. Comp. Psychol. 112, 56–63. 10.1037/0735-7036.112.1.55 9528114

[B83] Wundrum I. J. (1986). Cortical motor asymmetries and hominid feeding strategies. Hum. Evol. 1, 183–188. 10.1007/BF02437494

[B84] Zhao D. Gao X. Li B. (2010). Hand preference for spontaneously unimanual and bimanual coordinated tasks in wild Sichuan snub-nosed monkeys: implications for hemispheric specialization. Behav. Brain Res. 208, 85–89. 10.1016/j.bbr.2009.11.011 19914301

[B85] Zhao D. Hopkins W. D. Li B. (2012). Handedness in nature: first evidence of manual laterality on bimanual coordinated tube task in wild primates. Am. J. Phys. Anthropol. 148, 36–44. 10.1002/ajpa.22038 22410843 PMC3342595

